# Differential effects of child-appropriate music styles on executive function in preschool children: a longitudinal experimental study

**DOI:** 10.3389/fpsyg.2026.1779286

**Published:** 2026-03-18

**Authors:** Zaihao Wu, Yi Li, Keping Yan, Pingyang Xu, Cheng Yao, Diyue Zhong, Fang Wang

**Affiliations:** 1 Xihua University, Chengdu, China; 2 Lijiang Normal University, Lijiang, China; 3 Dianchi College, Kunming, China; 4 Jinzhou Medical University, Jinzhou, China; 5 Shinawatra University, Pathumthani, Thailand; 6 Batangas State University, Batangas, Philippines

**Keywords:** executive function, inhibitory control, music styles, preschool children, working memory

## Abstract

**Background:**

Executive function is a key capacity underpinning preschool children’s school readiness and self-regulation. Musical activities may facilitate executive function; however, direct, within-study comparisons of multiple child-appropriate musical styles remain limited. This study compared the effects of six musical styles (pop, jazz, Latin, electronic dance music, nursery rhymes, and folk/country) on preschoolers’ inhibitory control, working memory, and cognitive flexibility to assess their relative suitability.

**Methods:**

A longitudinal randomized controlled design was employed. A total of 115 children aged 4–5 years from a kindergarten in Chengdu were randomly assigned to six music-style groups or a control group. Over 4 weeks, the experimental groups participated in a brief daily classroom-based activity (10 min/weekday) that combined instrumental music exposure with rhythm-based clapping synchronized to the assigned style, whereas the control group engaged in quiet reading or drawing. Executive function was assessed at pretest, posttest, and one-month follow-up using the Peg Tapping Task, Rotating Can Task, and Dimensional Change Card Sort Task. Data were analyzed in SPSS using mixed-design ANOVAs, with Bonferroni-adjusted *post hoc* tests.

**Results:**

No significant between-group differences were observed at baseline. Following the intervention, the music condition demonstrated greater improvements than the control group in inhibitory control and working memory at posttest and follow-up. Improvements in cognitive flexibility were comparatively modest. When musical styles were examined separately, statistically robust advantages over the control condition were primarily concentrated in the Latin music group, whereas other styles showed improvement trends that did not consistently remain significant after correction for multiple comparisons. Maintenance of gains at follow-up was most evident in the Latin group.

**Conclusion:**

Music-based intervention can facilitate executive-function development in preschool children, particularly in inhibitory control and working memory. However, style-related differences were selective rather than forming a stable hierarchical gradient across genres. Latin music showed the most consistent and sustained advantages within the present design, suggesting that rhythmic engagement may support certain components of executive function. Further research is needed to isolate the specific musical parameters underlying these differences.

## Introduction

Executive function refers to a set of capacities that support the planning, monitoring, and regulation of cognition and behavior during goal-directed activities. It is typically conceptualized as inhibitory control, working memory, and cognitive flexibility (Schäfer et al., 2024; Escobar and Espinoza, 2025). During the preschool period, executive function develops rapidly and is closely associated with maturation of prefrontal-centered cognitive control networks, including increased myelination and strengthened functional connectivity. These neurodevelopmental changes enable children to inhibit impulses, maintain goals, and adapt behavior more flexibly and efficiently (Fiske and Holmboe, 2019; Jurado and Rosselli, 2007). A substantial body of research indicates that executive function is associated with adaptive outcomes, including school readiness, academic achievement, self-regulation, peer relationships, and classroom engagement. It is also associated with sustained attention, rule compliance, problem solving, and the use of learning strategies, and it predicts later developmental trajectories (Cao and Yan, 2024; Gestsdottir et al., 2023; Pumyoch and Srikoon, 2024; Marulis and Nelson, 2021). Therefore, identifying effective approaches to promote executive function in preschool children remains a priority.

Musical activities are closely associated with executive-function development and are considered feasible and ecologically valid interventions. Systematic reviews and meta-analyses indicate that music training is associated with improvements in inhibitory control, working memory, and cognitive flexibility in preschool children (Lu et al., 2025). Music is a highly temporally structured stimulus: rhythm, meter, phrasing, and formal sections provide an ongoing temporal framework; melodic and harmonic progressions create predictable patterns; and variations and accents prompt ongoing expectation updating and response adjustment. When children engage in musical activities (e.g., rhythm discrimination, movement following, clapping/stepping, or music games), they must maintain goals over time, inhibit impulsive responses, update rules, and adjust behavior in response to musical cues, thereby engaging executive-function processes. Empirical studies provide convergent support. For example, Shen et al. (2019) implemented a music-training program for preschool children and reported that improvements in executive function were maintained at follow-up. Similarly, a randomized controlled trial by Williams et al. (2023) found that a music program improved preschool children’s self-regulation and executive-function–related outcomes.

Differences in musical style can alter perceptual and emotional experiences and, in turn, modulate the recruitment of executive functions. Rhythmic structure, stylistic features, and improvisational demands may jointly influence temporal control, attentional regulation, and cognitive flexibility. Music with salient rhythmic profiles and relatively complex structures (e.g., jazz, electronic music) may strengthen time perception and motor coordination, thereby improving executive-control efficiency (Danielsen et al., 2022). Music with distinctive cultural or affective qualities (e.g., gospel, indigenous/local music) may indirectly influence executive-task performance through emotional arousal and motivational activation (Balogun et al., 2022). In addition, music activities with stronger improvisational components may be more likely to engage prefrontal and attentional control networks, facilitating cognitive flexibility and executive-function recovery (Koshimori and Thaut, 2019). Training level and modality also shape these pathways: highly trained musicians show advantages in response speed and task switching (Passarotto et al., 2023), whereas improvisers exhibit stronger cognitive control, potentially reflecting sustained monitoring and flexible adjustment (Hadar and Rabinowitch, 2023). Moreover, Chen et al. (2021) reported that inhibitory control and working memory are optimized when musical rhythm is synchronized with an individual’s heart rate, suggesting that rhythmic features and physiological synchrony moderate executive-function enhancement. From a neurobiological perspective, musical performance involves coordination among semantic, motor, and emotional memory systems, providing a physiological basis for style-related differences in executive control (Dymnikowa et al., 2020; Watcharinrat et al., 2024).

Although existing research supports the role of musical activities in promoting executive function, important gaps persist. Prior work has not sufficiently differentiated musical styles when characterizing musical stimulation, even though styles differ systematically in rhythmic complexity, structural variation, groove, and emotional arousal. These differences may impose distinct cognitive loads and regulatory demands and therefore should not be treated as interchangeable. Building on this premise, we define “child-appropriate music” as materials with safe sound-pressure levels, age-appropriate emotional content, predictable structure, and high potential for synchronized participation in preschool settings. To reduce language-processing demands, we prioritized instrumental versions. Nevertheless, direct comparisons of multiple child-appropriate musical styles within a single framework remain limited, leaving the practical question, “which music is most suitable for children?,” unresolved. Evidence remains limited regarding how music intervention influences different components of executive function and whether these effects persist over time. The present study used a 7 (group) × 3 (time) longitudinal design to examine changes in inhibitory control, working memory, and cognitive flexibility following music intervention in preschool children. In addition to evaluating overall intervention effects relative to a control condition, exploratory analyses examined whether patterns of change varied across musical styles. This study addressed the following research questions:

(1) Does a music-based intervention improve preschool children’s executive function compared with a control condition?
(2) To what extent do executive function outcomes vary across different musical styles?
(3) Are intervention-related changes maintained at follow-up?

## Methods

### 1.1 Participants

A convenience-sampling approach was used to recruit 119 children aged 4 to 5 years from a kindergarten in Chengdu. Based on health records and parent questionnaires, four children were excluded: two with a history of hearing impairment or neurodevelopmental conditions and two with incomplete data due to prolonged absence. The final sample comprised 115 children (mean age = 59.02 months, SD = 3.47). All participants were typically developing native Mandarin speakers with normal vision and hearing. None had previously received systematic instrumental or vocal training, and none had participated in similar music-intervention studies. Random assignment was conducted using a computer-generated random-number table prepared by an independent researcher who was not involved in participant recruitment, intervention delivery, or outcome assessment. Children were enrolled by research staff and subsequently assigned to groups according to the pre-generated allocation list. Allocation was concealed until assignment to prevent selection bias.

Participants were randomly assigned to one of seven groups: pop, jazz, Latin, electronic dance music, nursery rhymes, folk/country, or a control group, labeled E1 to E6 and C1, respectively (see Table 1 for details). No statistically significant between-group differences were found in age or sex ratio (*p* > 0.05). Outcome assessments were conducted by trained research assistants who were blinded to group allocation. Standardized administration procedures were followed to minimize potential observer bias. The study was conducted in accordance with the principles of the Declaration of Helsinki. Prior to implementation, informed consent was obtained from kindergarten administrators and parents or guardians, and assent was obtained from the children. The research protocol was approved by the ethics committee of X University (Ref. No. XHLL-K016).

**Table 1:** Participant characteristics.

Group	Music genre	*n*	Boys (n)	Girls (n)	Age in months (*M* ± SD)
E1	Pop	16	8	8	59.31 ± 3.25
E2	Jazz	16	7	9	58.75 ± 3.63
E3	Latin	16	7	9	59.06 ± 3.34
E4	Electronic dance music	16	8	8	59.00 ± 3.57
E5	Nursery rhymes	17	8	9	58.94 ± 3.53
E6	Folk/Country	17	8	9	58.94 ± 3.41
C1	Control	17	8	9	59.10 ± 3.77

### 1.2 Materials

Prior to the formal experiment, approximately 21 parents, teachers, and music experts reviewed candidate pieces to confirm that each category matched the intended stylistic characteristics and to exclude pieces for which reviewers expressed substantial disagreement. Ultimately, 10 to 12 instrumental pieces were selected for each of the six musical styles, and total duration, primary timbre, and recording quality were kept as consistent as possible across styles.

To minimize language-related processing demands that could draw on attentional and working-memory resources (Souza and Barbosa, 2023), all musical materials were instrumental, delivered via the same Bluetooth speaker (Xiaomi LX06) at a standardized 60–65 dB (measured with a digital sound level meter). Across the six styles, key variables were controlled for consistency: each excerpt was 2–3 min, recording quality was unified at 44.1 kHz/16-bit, and primary timbre was restricted to piano or strings (to avoid engagement variability from timbre). Styles differed primarily in tempo (95–138 BPM), metrical salience, and emotional arousal (indexed by dynamic range). To address the lack of objective acoustic data in prior research, we supplemented characterization with quantified features for each style, analyzed via Praat (v6.3.07) and Audacity (v3.4.2). These objective descriptors are presented in Table 2.

**Table 2:** Objective acoustic features of musical materials across styles.

Musical style	Tempo (BPM, *M* ± SD)	Beat salience (relative units)	Dynamic range (dB, *M* ± SD)	Rhythmic regularity (CV of inter-beat intervals)
Pop (E1)	98 ~ 106	Moderate (0.62)	8.2 ~ 9.5	0.11
Jazz (E2)	108 ~ 120	Moderate-High (0.71)	10.5 ~ 12.2	0.18
Latin (E3)	128 ~ 138	High (0.83)	9.8 ~ 11.3	0.08
Electronic Dance (E4)	125 ~ 130	High (0.80)	7.6 ~ 8.7	0.06
Nursery Rhymes (E5)	95 ~ 101	Low-Moderate (0.58)	6.9 ~ 7.9	0.1
Folk/Country (E6)	102 ~ 111	Moderate (0.65)	9.2 ~ 10.6	0.13

Beat salience measures periodicity strength in 1–4 Hz (for preschooler motor synchronization); rhythmic regularity is the CV of inter-beat intervals (lower = more predictable); dynamic range reflects intensity variation (a proxy for arousal); “~” indicates the value range.

### 1.3 Intervention procedure

The intervention was conducted in a quiet multipurpose activity room within the kindergarten. One week prior to the intervention, the research team coordinated the schedule with classroom teachers and introduced the “Listening to Music and Hand Clapping Game” using pictures and demonstrations to familiarize children with the activity. Groups E1 to E6 participated in music listening and rhythm-based clapping activities using instrumental excerpts from the assigned musical style. Each session lasted approximately 10 min and was conducted on weekday mornings over four consecutive weeks. Attendance was recorded at each session, and children who were absent for more than 10% of sessions were excluded from analysis. This ensured adequate exposure consistency across participants.

The intervention was delivered five times per week, resulting in approximately 200 min of total exposure. This cumulative dosage is comparable to prior studies summarized in recent meta-analyses, which have reported effective interventions using, for example, 20-min sessions delivered three times per week or 40-min sessions delivered twice per week (Lu et al., 2025). During each session, three to four instrumental excerpts were played via a Bluetooth speaker (Xiaomi LX06) at a standardized sound level of 60–65 dB (measured using a digital sound level meter), including approximately 1 min of fade-in and 1 min of fade-out.

To ensure fidelity of implementation, all sessions followed a standardized protocol specifying timing, volume range, clapping prompts, and activity structure. The same research assistants and classroom teachers supervised sessions across groups to maintain consistency. Periodic supervision by the principal investigator verified adherence to the protocol. During playback, children were encouraged to clap along with the musical beat intermittently while remaining free to adopt a comfortable posture. Researchers and teachers guided the activity to ensure consistent implementation across sessions while allowing natural participation. These rhythm-based clapping activities required children to attend to the temporal structure of the music and coordinate their responses accordingly, thereby ensuring active engagement with the auditory stimuli.

For example, in Session 2 of the pop music group (90–110 BPM), children listened to instrumental excerpts and clapped along with the musical beat when prompted by the researchers. Clapping occurred intermittently and was naturally integrated within the listening activity. Similarly, in Session 5 of the Latin music group (120–140 BPM), children listened to instrumental excerpts and intermittently clapped in synchrony with the faster and more salient rhythmic structure. These rhythm-based activities were consistent across groups, with only the musical style and tempo differing.

Children in the control group (C1) remained in a quiet classroom during the same time period and engaged in self-directed reading or simple drawing. No music was played, and no rhythm-based activities were organized.

### Measures

#### Peg Tapping Task

The Peg Tapping Task, originally proposed by Luria (1966), was used to assess inhibitory control in preschool children. This task requires children to suppress the tendency to imitate the experimenter’s taps. Prior to testing, children demonstrated understanding of two rules: (1) when the experimenter taps once, the child taps twice; and (2) when the experimenter taps twice, the child taps once. A practice phase followed. The experimenter demonstrated the task using a wooden peg and provided praise or corrective feedback as needed. Practice trials continued until the child could reliably follow both rules, with no preset limit.

Following Bowmer et al. (2018), the experimenter administered 16 trials in pseudorandom order. No feedback was provided during testing. Each correct response was scored as 1 and each incorrect response as 0, yielding a total score ranging from 0 to 16. If a child was unable to understand the rules or complete the task, a score of minus 1 was assigned. Common errors included: (1) following only one rule, (2) tapping repeatedly regardless of the stimulus, or (3) directly imitating the experimenter. The task has demonstrated good test retest reliability (*r* = 0.80) (Lipsey et al., 2017).

#### Rotating Can Task

The Rotating Can Task was used to measure working memory in preschool children (Hughes and Ensor, 2005). Following Ilari et al. (2021), eight opaque paper cups were placed on a table, each marked with a distinct symbol (e.g., circles, triangles) for identification. Children placed one colored ball under each of six cups, leaving the remaining two cups empty. Children were then blindfolded, and the experimenter shuffled the cups out of view. The child then lifted the cups one by one to locate the balls, aiming to find all six balls in as few attempts as possible. Each cup lift counted as one attempt, and lifting an empty cup counted as one error. A maximum of 16 attempts was allowed, and the task ended once all six balls were found. The score was calculated as 16 minus the number of errors, with higher scores indicating better working-memory performance.

#### Dimensional Change Card Sort Task

The Dimensional Change Card Sort Task (Zelazo, 2006) was used to assess cognitive flexibility. Two sorting trays were used, each displaying two fixed target cards: a yellow star and a green circle. The experimenter pointed to each target card and verbally labeled it.

The task consisted of two phases: a pre-switch phase and a post-switch phase. In the first phase, the experimenter demonstrated sorting by color (Ilari et al., 2021). Children placed yellow cards next to the yellow target card and green cards next to the green target card. After the demonstration, children sorted six cards according to color. In the second phase, the sorting rule was changed. The experimenter instructed the child to sort by shape, stating, “Stars go here, and circles go here.” Children then sorted six new cards according to shape.

Children who correctly sorted five or more cards in the post-switch phase completed the border version of the Dimensional Change Card Sort Task. In this version, children selected the sorting rule based on the presence of a black border: bordered cards were sorted by color, and unbordered cards were sorted by shape. Cards were shuffled randomly, and the order of dimension presentation was counterbalanced. Scoring followed Zelazo’s (2006) recommendations. In the pre-switch phase, 0 to 4 correct responses received 0 points and 5 to 6 received 1 point. In the post-switch phase, 0 to 4 correct responses received 1 point and 5 to 6 received 2 points. In the border version, fewer than 9 correct responses received 2 points, and 9 or more received 3 points.

### Data collection

Executive function was assessed at three time points: pretest (T1), posttest (T2), and follow-up (T3). The study was conducted in a single kindergarten with 7 classrooms, and randomization was not stratified by classroom. All assessments were administered individually in a dedicated quiet room (separate from regular classrooms and intervention spaces) by trained research assistants blinded to group allocation. Each one-on-one assessment lasted ~20 min, following standardized procedures (uniform task instructions, scoring criteria, and environmental controls). After T1, children returned to their original classroom for quiet activities prior to intervention. T2 was conducted 3–5 min post-final intervention/control session, and T3 (1-month post-intervention) employed identical measures and protocols. Follow-up completion rate was 100%. Cross-group contamination was minimized by scheduling intervention/control sessions simultaneously in separate rooms (5 for experimental groups, 1 for control) with no inter-room participant overlap. Classroom teachers only assisted with participant escort, with no involvement in assessments.

### Data analysis

This study was not prospectively registered, but research questions and statistical plans were pre-established. Primary analyses focused on the group × time interaction (music vs. control) for total executive-function scores (T1–T2), with maintenance effects (T1–T3), subcomponent analyses (inhibition, working memory, cognitive flexibility), and style comparisons (E1–E6) as secondary/exploratory. Data were analyzed via SPSS 26.00. Preliminary analyses confirmed normality and no baseline group differences. Mixed-design ANOVAs (group as between-subject, time [T1–T3] as within-subject) with Greenhouse–Geisser corrections and Bonferroni-adjusted *post hoc* tests were used. Effect sizes are reported as ηp^2^ (*p* < 0.05). A sensitivity analysis (mixed models with classroom as a random effect) for key outcomes confirmed consistent results with original ANOVAs (significant group × time interactions, *p* < 0.01), supporting findings’ robustness.

### Rigor

Executive-function assessments were administered by trained research assistants who were not involved in delivering the intervention sessions. At the time of testing, assessors were unaware of participants’ group allocation to the extent feasible within the school setting. Standardized administration and scoring procedures were followed to minimize subjective bias, and data were analyzed using anonymized participant codes.

## Results

Preliminary analyses confirmed no significant baseline differences in inhibitory control, working memory, or cognitive flexibility between music intervention and control groups (all *p*s > 0.05), confirming group comparability pre-intervention. Primary mixed-design ANOVAs examined overall music intervention effects, with exploratory analyses further assessing musical style differences. Means and standard deviations for the experimental and control groups at different time points are presented in Table 3, and the detailed results of each executive function component across all subgroups are shown in Table 4.

**Table 3:** Descriptive statistics and ANOVA results of executive function tasks between experimental and control groups.

Variables	Mean ± SD		ANOVA		
	Experimental group (E1-E6)	Control group (C1)	*F*	*p*	ηp^2^
Peg Tapping Task					
Pre-test	6.05 ± 1.30	5.82 ± 1.19	0.457	0.5	0.004
Post-test	7.13 ± 1.23	5.94 ± 1.09	13.981	<0.001	0.109
Follow-up test	7.24 ± 1.16	6.00 ± 1.00	17.14	<0.001	0.13
Rotating Can Task					
Pre-test	7.99 ± 2.12	8.12 ± 2.12	0.053	0.819	0
Post-test	9.76 ± 1.81	8.29 ± 1.96	9.204	0.003	0.075
Follow-up test	9.91 ± 1.66	8.35 ± 1.87	12.219	<0.001	0.098
Dimensional Change Card Sort Task					
Pre-test	1.16 ± 0.76	1.12 ± 0.70	0.054	0.817	0
Post-test	1.50 ± 0.60	1.18 ± 0.64	4.182	0.043	0.036
Follow-up test	1.59 ± 0.59	1.18 ± 0.64	7.036	0.009	0.058

**Table 4:** Descriptive statistics and group comparisons for inhibitory control, working memory, and cognitive flexibility.

Variables	Mean ± SD							ANOVA		
	E1	E2	E3	E4	E5	E6	C1	*F*	*p*	ηp^2^
Peg Tapping Task										
Pre-test	5.81 ± 1.60	6.38 ± 0.96	6.25 ± 1.24	5.94 ± 1.18	6.00 ± 1.50	5.94 ± 1.30	5.82 ± 1.19	0.439	0.851	0.024
Post-test	7.00 ± 1.59	7.13 ± 0.96	7.50 ± 1.21	7.06 ± 1.12	7.24 ± 1.25	6.88 ± 1.27	5.94 ± 1.09	2.696	0.018	0.130
Follow-up test	7.13 ± 1.45	7.19 ± 0.83	7.63 ± 1.15	7.19 ± 1.11	7.29 ± 1.21	7.00 ± 1.17	6.00 ± 1.00	3.270	0.005	0.154
Rotating Can Task										
Pre-test	8.00 ± 2.22	8.06 ± 2.17	8.06 ± 2.17	7.81 ± 2.29	8.06 ± 2.11	7.94 ± 2.11	8.12 ± 2.12	0.037	1	0.002
Post-test	9.69 ± 1.89	9.88 ± 1.93	10.13 ± 1.63	9.75 ± 2.08	9.71 ± 1.79	9.41 ± 1.73	8.29 ± 1.96	1.702	0.127	0.086
Follow-up test	9.81 ± 1.72	10.06 ± 1.73	10.44 ± 1.46	9.88 ± 1.89	9.82 ± 1.59	9.47 ± 1.66	8.35 ± 1.87	2.477	0.028	0.121
Dimensional Change Card Sort Task										
Pre-test	1.25 ± 0.86	1.25 ± 0.93	1.13 ± 0.72	1.06 ± 0.68	1.12 ± 0.70	1.18 ± 0.73	1.12 ± 0.70	0.142	0.990	0.008
Post-test	1.50 ± 0.63	1.56 ± 0.73	1.63 ± 0.50	1.50 ± 0.52	1.41 ± 0.62	1.41 ± 0.62	1.18 ± 0.64	0.933	0.475	0.049
Follow-up test	1.63 ± 0.62	1.63 ± 0.72	1.81 ± 0.40	1.63 ± 0.50	1.47 ± 0.62	1.41 ± 0.62	1.18 ± 0.64	1.932	0.082	0.097

### Peg Tapping Task

**Figure 1:**
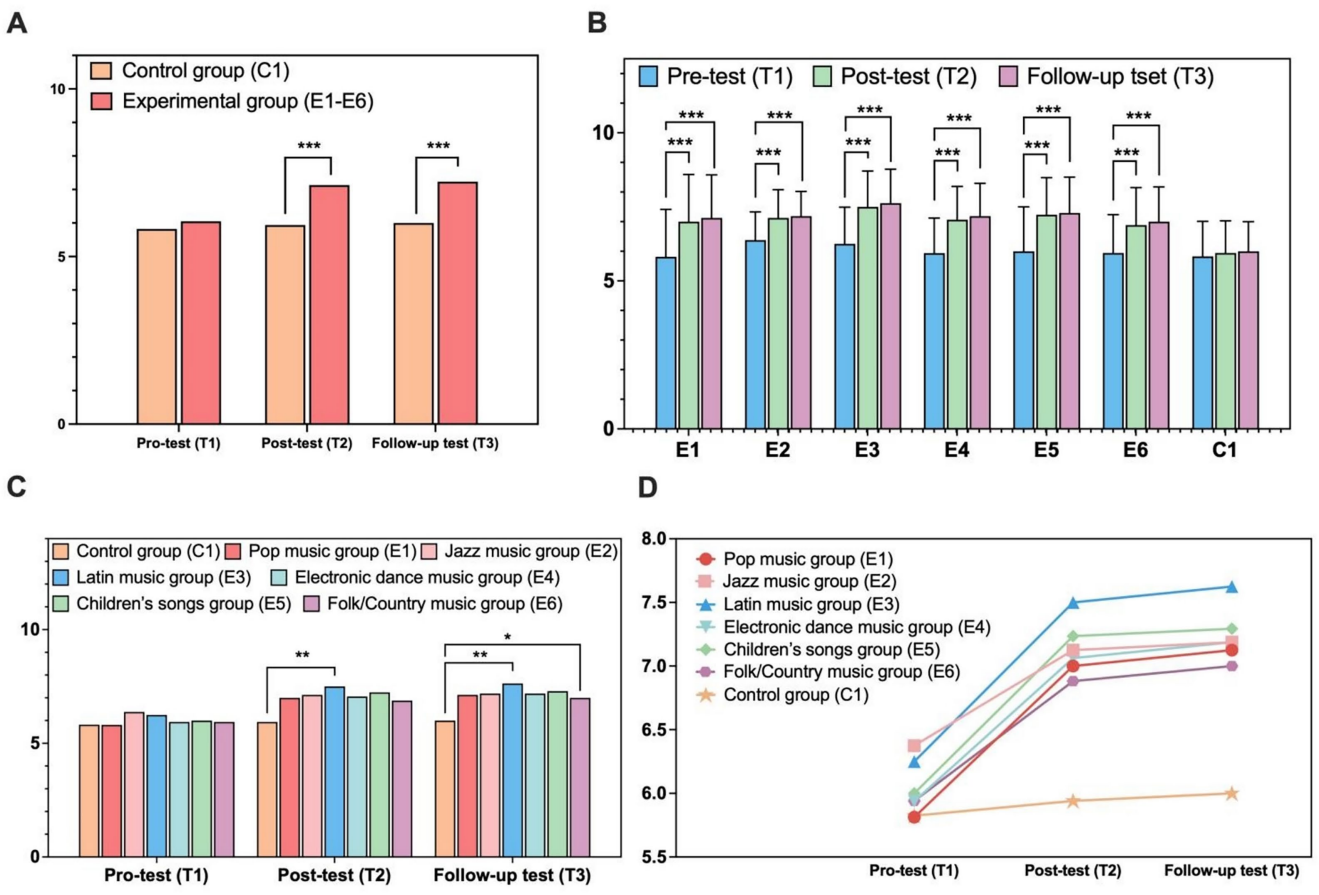
Effects of music interventions on preschoolers’ inhibitory control. **(A)** Overall comparison between experimental and control groups. **(B)** Temporal changes across all subgroups. **(C)** Group-wise scores at each time point. **(D)** Longitudinal growth trajectories of each subgroup. ^*^p < 0.05; ^**^p < 0.01; ^***^p < 0.001.

All participants successfully completed the Peg Tapping Task at T1–T2 with no missing scores, and no ceiling or floor effects were observed for any executive function task across all time points. A 2 (group: music intervention vs. control) × 3 (time: T1, T2, T3) mixed-design ANOVA was conducted to examine the intervention effect on inhibitory control. Greenhouse–Geisser corrections were applied due to sphericity violation [*χ*^2^(2) = 28.205, *p* < 0.001, *ε* = 0.818], yielding a significant main effect of time {*F*(1.636, 184.848) = 81.958, *p* < 0.001, ηp^2^ = 0.420, 95% CI [0.33, 0.50]}, a significant main effect of group {*F*(1, 113) = 8.090, *p* = 0.005, ηp^2^ = 0.067, 95% CI [0.01, 0.15]}, and a significant group × time interaction {*F*(1.636, 184.848) = 48.114, *p* < 0.001, ηp^2^ = 0.298, 95% CI [0.21, 0.37]}. Bonferroni-adjusted *post hoc* comparisons revealed no baseline group difference (*p* = 0.500), but the experimental group scored significantly higher than the control group at posttest and follow-up (both *p*s < 0.001, Cohen’s *d* = 0.78–0.81; see Table 3 and Figure 1A). Within-group analyses showed significant improvements across all time points in the experimental group (all *p*s < 0.001), whereas no significant changes were found in the control group (all *p*s ≥ 0.36).

To explore variability in intervention effects across musical styles, a 7 (group) × 3 (time) mixed-design ANOVA was conducted. Mauchly’s test indicated sphericity violation [*χ*^2^(2) = 20.271, *p* < 0.001], and Greenhouse–Geisser corrections were applied (*ε* = 0.853). Analyses revealed a large significant main effect of time {*F*(1.706, 184.209) = 414.254, *p* < 0.001, ηp^2^ = 0.79, 95% CI [0.73, 0.83]} and a large significant group × time interaction {*F*(10.234, 184.209) = 10.623, *p* < 0.001, ηp^2^ = 0.37, 95% CI [0.22, 0.48]}; the main effect of group was non-significant {*F*(6, 108) = 1.655, *p* = 0.139, ηp^2^ = 0.09, 95% CI [0.00, 0.20]}.

Across all groups, inhibitory control scores increased significantly from T1 to T2 and T1 to T3 (both *p*s < 0.001, Cohen’s *d* = 0.74–0.82), with a small additional significant rise from T2 to T3 (*p* = 0.007, Cohen’s *d* = 0.08; see Table 4). All experimental subgroups (E1–E6) showed large significant improvements from T1 to T2 and T1 to T3 (all *p*s < 0.001, Cohen’s *d* = 0.65–1.05), while the control group had no significant changes across time points (all *p*s ≥ 0.413, Cohen’s *d* = 0.06–0.14; see Figure 1B).

Between-group comparisons showed no baseline differences at T1 [*F*(6,108) = 0.439, *p* = 0.851, ηp^2^ = 0.02], but significant medium effects at T2 [*F*(6,108) = 2.696, *p* = 0.018, ηp^2^ = 0.13] and large effects at T3 [*F*(6,108) = 3.270, *p* = 0.005, ηp^2^ = 0.16; 95% CIs for all analyses see text]. Bonferroni-adjusted pairwise comparisons indicated E3 scored significantly higher than the control at T2 and T3 (both *p*s < 0.01, Cohen’s *d* = 1.48–1.55), and E5 differed significantly from the control at T3 (*p* = 0.028, Cohen’s *d* = 1.22; see Figure 1C). Other experimental subgroups showed non-significant improvement trends after Bonferroni correction (all *p*s > 0.05) with small-to-medium effect sizes (Cohen’s *d* = 0.31–0.87). The longitudinal trajectories of each group are shown in Figure 1D.

### Rotating Can Task

**Figure 2:**
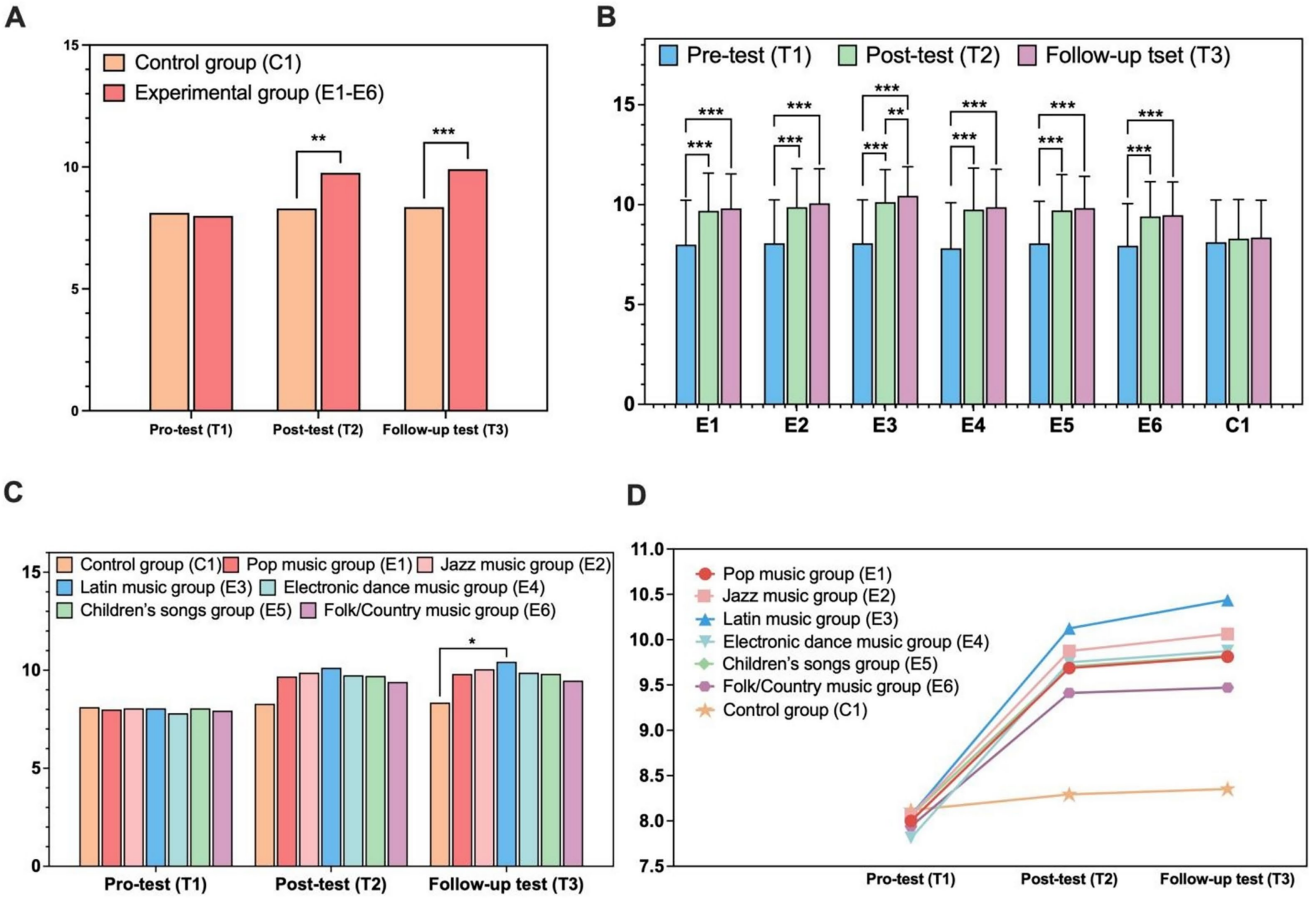
Effects of music interventions on preschoolers’ working memory. **(A)** Overall group comparison; **(B)** working memory across subgroups over time; **(C)** temporal changes across music genres; **(D)** longitudinal growth trajectories. ^*^p < 0.05; ^**^p < 0.01; ^***^p < 0.001.

Music experimental groups exhibited greater working memory gains over time than the control group. Mauchly’s test indicated sphericity violation [*χ*^2^(2) = 123.861, *p* < 0.001], with Greenhouse–Geisser corrections applied (*ε* = 0.599). A mixed-design ANOVA revealed a large significant main effect of time {*F*(1.198, 135.403) = 77.692, *p* < 0.001, ηp^2^ = 0.57, 95% CI [0.46, 0.66]}, a large significant group × time interaction {*F*(1.198, 135.403) = 49.316, *p* < 0.001, ηp^2^ = 0.31, 95% CI [0.19, 0.42]}, and a small significant main effect of group {*F*(1, 113) = 3.948, *p* = 0.049, ηp^2^ = 0.03, 95% CI [0.00, 0.12]}. Bonferroni-adjusted comparisons found no baseline group difference (*p* = 0.819, *d* = −0.06; see Table 3 and Figure 2A), but the experimental group scored significantly higher at posttest and follow-up (both *p*s < 0.01, *d* = 0.79–0.86). The experimental group showed consistent significant improvements across all time points (all *p*s < 0.001, *d* = 0.10–1.04), whereas the control group had no meaningful changes (all *p*s ≥ 0.577, *d* = 0.09–0.13).

We further probed performance variations in working memory across the seven musical style subgroups using a 7 × 3 mixed-design ANOVA. Greenhouse–Geisser correction was implemented to address sphericity violation [*χ*^2^(2) = 113.757, *p* < 0.001, *ε* = 0.604]. Analyses revealed a robust main effect of time, with working memory scores increasing significantly across assessments {*F*(1.209, 130.543) = 388.940, *p* < 0.001, ηp^2^ = 0.78, 95% CI [0.71, 0.83]}. A significant group × time interaction was also observed {*F*(7.252, 130.543) = 9.605, *p* < 0.001, ηp^2^ = 0.35, 95% CI [0.22, 0.45]}, indicating the magnitude of working memory improvement differed by subgroup. No overall between-group differences were detected when scores were averaged across time points {*F*(6, 108) = 0.791, *p* = 0.579, ηp^2^ = 0.04, 95% CI [0.00, 0.12]}.

**Figure 3:**
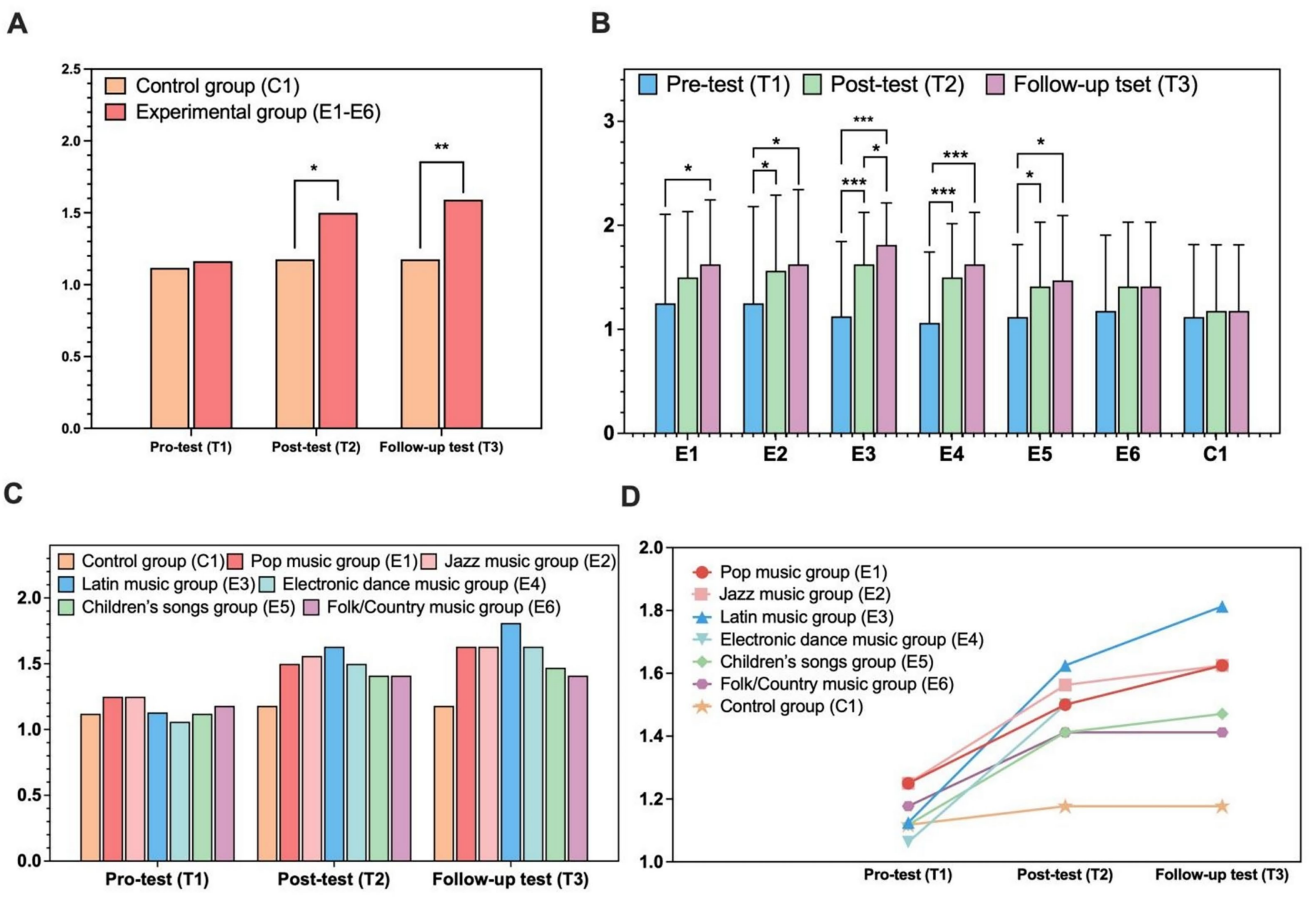
Effects of music interventions on preschoolers’ cognitive flexibility. **(A)** Overall group comparison. **(B)** Cognitive flexibility across subgroups over time. **(C)** Temporal changes across music genres. **(D)** Longitudinal growth trajectories. ^*^p < 0.05; ^**^p < 0.01; ^***^p < 0.001.

All experimental subgroups demonstrated significant working memory gains from baseline to posttest and follow-up (all *p*s < 0.001, Cohen’s *d* = 0.76–1.12; see Figure 2B), with only the E3 subgroup showing additional significant improvements from posttest to follow-up (*p* = 0.001, *d* = 0.35; see Table 4). The control group exhibited no significant changes in working memory across all assessments (all *p*s ≥ 0.567, *d* = 0.06–0.12; see Figure 2B). While no baseline between-group differences were found [*F*(6, 108) = 0.037, *p* = 0.999], significant subgroup differences emerged at the follow-up time point {*F*(6, 108) = 2.477, *p* = 0.028, ηp^2^ = 0.12, 95% CI [0.02, 0.23]}. Bonferroni-adjusted pairwise comparisons confirmed that the E3 subgroup scored significantly higher than the control group at follow-up (*p* = 0.014, *d* = 1.15; see Figure 2C); improvements in other experimental subgroups were non-significant after correction (all *p*s > 0.05, *d* = 0.42–0.78; see Figure 3C), with only small-to-medium effect sizes observed. The longitudinal trajectories of each group are shown in Figure 2D.

### Dimensional Change Card Sort Task

Cognitive flexibility showed gradual improvement over the study, with change magnitudes varying by group. Mauchly’s test detected sphericity violation [*χ*^2^(2) = 60.326, *p* < 0.001], and Greenhouse–Geisser corrections were applied (*ε* = 0.706). A mixed-design ANOVA revealed a medium significant main effect of time {*F*(1.412, 159.554) = 10.636, *p* < 0.001, ηp^2^ = 0.09, 95% CI [0.02, 0.18]} and a small significant group × time interaction {*F*(1.412, 159.554) = 5.877, *p* = 0.009, ηp^2^ = 0.05, 95% CI [0.01, 0.12]}, which indicated divergent change trajectories between the two groups. No overall main effect of group was found {*F*(1, 113) = 2.717, *p* = 0.102, ηp^2^ = 0.02, 95% CI [0.00, 0.09]}, and the music and control groups performed similarly at baseline (*p* = 0.817, *d* = 0.06; see Figure 3A).

Bonferroni-adjusted comparisons indicated the music group had significantly higher cognitive flexibility at posttest and follow-up (both *p*s < 0.05, *d* = 0.54–0.70). The music group also exhibited significant within-group gains from baseline to posttest and follow-up (both *p*s < 0.001, *d* = 0.56–0.71), whereas the control group showed no meaningful changes across all time points (all *p* ≥ 0.866, *d* = 0.03–0.05).

Gains in cognitive flexibility differed across the six experimental subgroups, with scores increasing overall from baseline to follow-up. Greenhouse–Geisser corrected analyses identified a large significant main effect of time {*F*(1.429, 154.283) = 50.580, *p* < 0.001, ηp^2^ = 0.318, 95% CI [0.201, 0.421]} and a significant group × time interaction {*F*(8.571, 154.283) = 2.058, *p* = 0.039, ηp^2^ = 0.104, 95% CI [0.006, 0.192]}; no overall performance differences were found across the seven groups when averaged across time {*F*(6, 108) = 0.662, *p* = 0.680, ηp^2^ = 0.036, 95% CI [0.000, 0.127]}.

Bonferroni-adjusted pairwise comparisons revealed several experimental subgroups, excluding E6, achieved significant baseline-to-follow-up improvements (all *p*s < 0.05, *d* = 0.52–1.18; see Table 4 and Figure 3B), with the Latin music subgroup demonstrating sustained sequential gains across all assessments (all *p*s < 0.05). The control group showed no significant changes at any time point (all *p*s ≥ 1.000, *d* = 0.03–0.06). Though several experimental subgroups had numerically higher posttest and follow-up scores than the control, all between-group differences were non-significant after multiple comparison correction (all *p*s ≥ 0.05; Figure 3C). The longitudinal trajectories of each group are shown in Figure 3D.

## Discussion

This study adopted a longitudinal experimental design to examine how different categories of child-appropriate music relate to preschool children’s executive function and whether any observed changes persist over time. In the present context, child-appropriate music refers to materials characterized by safe sound-pressure levels, age-appropriate emotional content, relatively predictable structure, and a strong potential for synchronized participation in preschool settings. To minimize language-related cognitive load and enhance interpretability of structural features, all musical materials were instrumental (Souza and Barbosa, 2023). High-intensity genres such as rock and heavy metal were excluded to ensure developmental suitability and to reduce excessive arousal that might introduce variability in engagement. Across the intervention period, children exposed to music demonstrated greater improvements than those in the control condition, particularly in inhibitory control and working memory. Gains in cognitive flexibility were present but comparatively smaller and less robust. When individual musical styles were examined, statistically reliable advantages over the control group were primarily concentrated in Latin music group across inhibitory control and working memory, with an additional follow-up effect observed for Nursery rhymes in inhibitory control. The isolated inhibitory-control effect in the nursery rhyme group may reflect its low-moderate beat salience and simple, repetitive rhythmic structure (see Table 2). This predictability may reinforce response inhibition through repeated timing practice, yet lacks sufficient updating demands to strengthen working memory (Suppalarkbunlue et al., 2023). Other styles showed improvement trends over time, but these differences did not consistently remain significant after correction for multiple comparisons. Follow-up findings further indicated that maintenance of gains was most evident in the style that demonstrated the strongest short-term effects, particularly Latin music group, whereas long-term differentiation from the control group was less consistent for other styles. Music intervention was beneficial overall, but the magnitude and durability of effects varied across executive-function components and were unevenly distributed across musical styles.

Regarding the first research question, the findings further support evidence that musical activities facilitate executive-function development in preschool children. After 4 weeks of intervention, children in the music condition showed significant improvements in inhibitory control and working memory relative to the control group, indicating that musical engagement may support goal maintenance and response regulation. This pattern is consistent with Shen et al. (2019), suggesting that the observed gains reflect systematic intervention effects rather than chance variation. By contrast, improvements in cognitive flexibility were comparatively modest. Although performance increased over time in the experimental group, the magnitude of group differences was weaker than that observed for inhibitory control and working memory. This aligns with Frischen et al. (2019), who proposed that rhythm-based training may preferentially strengthen inhibitory control, pointing to selective rather than uniform effects across executive-function components. By comparing multiple musical styles within a longitudinal framework, the present study extends prior work beyond single-intervention or short-term designs (Bowmer et al., 2018). The findings further suggest that characteristics such as rhythmic salience and emotional arousal may contribute to variation in intervention strength. In background-music contexts, low-arousal music has been associated with improved task performance, whereas highly arousing music linked to negative experience may impair memory processing (Hallam et al., 2002). However, such findings concern passive listening, whereas the present intervention required active rhythmic engagement, indicating that the influence of musical features may depend on functional context and mode of participation. These results support music as an ecologically valid avenue for cognitive intervention and highlight the need to clarify how specific musical characteristics relate to distinct executive-function components.

The results answer the second question, that differences across musical styles were present but not uniformly distributed. Statistically robust advantages over the control group were primarily concentrated in Latin music group, particularly in inhibitory control and working memory, whereas other styles showed improvement trends that were less consistent after correction for multiple comparisons. Rather than indicating a stable gradient across all genres, the pattern points to a more selective distribution of effects. One possible interpretation relates to rhythmic salience and temporal structure. Objective acoustic analysis (see Materials) revealed Latin music and electronic dance music shared high beat salience and low rhythmic irregularity, but differed in tempo and dynamic range. Notably, Latin’s slightly higher tempo and broader dynamic range may have enhanced sustained engagement, consistent with evidence that moderate acoustic contrast boosts preschoolers’ attentional investment (Quan et al., 2023), helping explain its consistent superiority. However, these features are confounded within genres (e.g., beat salience co-varies with tempo), precluding disentanglement of unique contributions.

Styles characterized by clearer beat structure and stronger synchrony demands may place greater emphasis on prediction, timing coordination, and response regulation, processes closely associated with executive control (Weineck et al., 2022). In this context, the relatively strong and sustained effects in the Latin music group suggest that rhythmic regularity and motor synchronization may be associated with executive-function gains; however, as these features were not independently manipulated, their causal role cannot be confirmed. At the same time, the absence of consistent superiority across all higher-tempo or higher-arousal styles suggests that tempo and arousal alone are unlikely to account for the observed differences. Indeed, tempo effects may not follow a simple linear pattern. Prior work has suggested a potential inverted-U relationship between tempo and inhibitory control, such that moderate rhythmic demands may optimize synchronization whereas excessive speed may increase cognitive load (Xiao et al., 2020). Similarly, very slow tempos may reduce arousal and sustained attention (Lin et al., 2023). The present results do not allow definitive conclusions regarding such mechanisms but are broadly compatible with the view that rhythmic characteristics interact with developmental capacity and task demands. Although it would be premature to draw prescriptive conclusions regarding genre selection, the findings tentatively suggest that musical styles emphasizing clear rhythmic structure and opportunities for synchronized engagement may be more likely to support inhibitory control and working memory in preschool contexts. Future research should experimentally isolate rhythmic density, tempo, and arousal to determine which specific musical parameters drive these differences.

Regarding the third research question, the facilitative effects of the music intervention were partly maintained after the intervention ended. At the overall level, the experimental group continued to outperform the control group at follow-up across executive-function components, although effects were stronger for inhibitory control and working memory. When musical styles were examined separately, maintenance of gains was most evident in Latin music group, whereas long-term differentiation from the control group was less consistent for other styles. These findings are broadly consistent with prior longitudinal evidence. Shen et al. (2019) reported that executive-function improvements following music training were maintained over a 12-week period, and Williams et al. (2020) similarly observed that rhythm- and movement-based self-regulation programs produced benefits that persisted for up to 1 year. Although the present results do not allow strong conclusions regarding specific musical parameters, the relatively stable effects observed in Latin music group are compatible with the view that rhythmic engagement and temporal coordination may contribute to longer-term consolidation of executive-function gains. In line with this interpretation, Lu et al. (2025) suggested that sustained transfer effects are more likely when rhythmic processing demands are sufficiently intensive and repeated over time. From a neurobiological perspective, it has been proposed that rhythmic synchronization may support multisensory integration and functional coordination within executive-control networks (Chan and Han, 2022). However, as the present study relied on behavioral measures, the underlying neural mechanisms remain speculative and warrant further investigation.

The findings further suggest that music intervention was associated with larger and more robust improvements in inhibitory control and working memory than in cognitive flexibility, pointing to component-specific rather than uniform effects. This dissociation may reflect both developmental timing and task demands. Between ages 4 and 5, inhibitory control and working memory undergo rapid maturation, whereas cognitive flexibility often develops more markedly after age 6 (Veraksa et al., 2020; Purpura et al., 2017), potentially making the former components more modifiable within this age range. From a process perspective, sustained inhibition and action updating during rhythmic synchronization have been associated with prefrontal–basal ganglia networks (Grahn and Rowe, 2009), which may help explain why inhibitory control appeared particularly responsive to music-based engagement. The repetitive and sequential structure of music may also tax working-memory processes by requiring maintenance of beat and rule representations. In contrast, cognitive flexibility relies more heavily on explicit rule switching and multi-strategy coordination. Although the present intervention incorporated rhythm-based clapping, it did not require systematic rule shifts or multidimensional task coordination, which may account for the comparatively smaller effects observed in cognitive flexibility. This pattern is consistent with Lu et al. (2025), who reported larger effects of music intervention on inhibitory control than on cognitive flexibility. Accordingly, when cognitive flexibility is the primary target, interventions may need to incorporate more interactive musical elements involving explicit rule changes, such as improvisation or multidimensional tasks (Norgaard et al., 2019), to increase demands on strategy shifting and cognitive updating.

Because the present study relied primarily on behavioral measures, the mechanisms and boundary conditions underlying the observed effects cannot be fully specified. It remains unclear whether improvements in executive function were driven by music-specific processes such as rhythmic synchronization and emotional arousal, or by more general factors including engagement and motivational activation. Similarly, the study design did not permit isolation of specific musical parameters (e.g., tempo, rhythmic density, and arousal level) or identification of dosage thresholds associated with maintenance and delayed effects. The comparatively smaller effects observed for the Dimensional Change Card Sort Task may reflect developmental timing, partial misalignment between the intervention activities and task demands, or limitations in measurement sensitivity. Future research incorporating process-level indicators, experimental manipulation of musical features, and, where feasible, neurophysiological measures would help clarify the mechanisms through which music-based interventions influence distinct components of executive function.

Several limitations should be considered. First, while the overall sample size supported detection of moderate intervention effects, small per-style samples limited sensitivity to subtle between-style differences, particularly for cognitive flexibility. Non-significant contrasts therefore should not be interpreted as equivalence. Restricting comparisons to six predefined categories further constrained stylistic breadth. Second, executive-function tasks were repeated across three time points using identical measures, introducing potential practice effects. Although the control group did not show comparable gains, repeated exposure may have contributed to follow-up improvements. As executive-function research typically reports stability rather than continued growth without booster sessions, maintenance effects should be interpreted cautiously. Future work could employ alternate task forms or extended follow-up intervals. Third, the relatively narrow age range and recruitment from a single kindergarten constrain generalizability. Replication across culturally and geographically diverse contexts, and among children with varying levels of prior musical exposure, would strengthen external validity. Finally, restricting materials to instrumental and developmentally appropriate styles reduced linguistic confounding but narrowed ecological scope. Including music with lyrics and a wider range of intensity levels in future research would clarify how linguistic and arousal factors interact with executive-function components.

## Conclusion

By directly comparing six categories of child-appropriate musical styles within a unified framework, the present study shows that music-based intervention can facilitate executive-function development in preschool children, particularly in inhibitory control and working memory. Improvements in cognitive flexibility were comparatively modest, indicating component-specific rather than uniform effects. Differences across musical styles were evident, but statistically robust advantages over the control condition were primarily concentrated in the Latin music group. Latin music showed consistent superiority in inhibitory control at posttest and follow-up, and maintained advantages in working memory at follow-up, whereas differentiation among the remaining genres was less consistent after correction for multiple comparisons. These findings suggest a selective concentration of effects rather than a stable hierarchical gradient across styles. Follow-up analyses further indicated that maintenance of gains was most clearly observed in the Latin music group, pointing to the possibility that rhythmic salience and synchronized engagement may contribute to sustained benefits. However, the present findings do not support definitive prescriptive rankings of musical genres. Under developmentally appropriate and safe conditions, rhythmic participation, exemplified here by Latin music, appears to be a promising avenue for supporting executive-function development, while future research is needed to isolate the specific musical parameters underlying these differences.

## Data Availability

The original contributions presented in the study are included in the article/supplementary material, further inquiries can be directed to the corresponding authors.
